# A Scoping Review of the Emergence of Novel Synthetic Opioids in Australian Drug Markets: What Does This Mean for Harm Reduction Responses?

**DOI:** 10.1111/dar.14079

**Published:** 2025-05-28

**Authors:** Emmanuel Mammoliti, Suzanne Nielsen, Amanda Roxburgh

**Affiliations:** ^1^ National Drug Research Institute Curtin University Perth Australia; ^2^ Disease Elimination Burnet Institute Melbourne Australia; ^3^ Monash Addiction Research Centre Monash University Melbourne Australia

**Keywords:** fentanyl analogues, harm reduction, nitazenes, novel synthetic opioids, potent synthetic opioids

## Abstract

**Issues:**

Rising synthetic opioid deaths in North America, and the emergence of nitazene harms has driven concern about novel synthetic opioids (NSO) in Australia. This scoping review aimed to map what is known about NSOs in Australia.

**Approach:**

Scoping review of peer‐reviewed and grey literature on NSO detections and harms. Criteria: studies describing NSO market indicators and harms in Australia.

**Key Findings:**

Overall, 912 peer‐reviewed studies were identified; 40 met criteria. Monitoring systems identified NSOs (e.g., β‐U10, nitazenes) for the first time in Australia. Nitazene toxicity appeared in emergency departments (*N* = 11, 2021‐onwards), two involved intentional consumption. NSOs for sale to Australia on cryptomarkets comprised < 1% of drug listings. Mortality studies identified fentanyl analogues (furanylfentanyl, acetylfentanyl) (*N* = 22, 2013–2021), and other NSO U‐47700; (*N* = 12, 2016–2021) deaths. Thirty‐three nitazene deaths were reported; 24 in Victoria (2021‐onwards); 7 in SA (2022‐onwards); two in NSW (2024). Australian Federal Police reported 47 nitazene seizures since July 2023. Fifty‐one drug alerts were identified (2019–2024); 18 (34%) involved NSOs; 12 (24%) for nitazenes. Overall, 12 alerts were for NSO‐contaminated stimulants; at least two fatal overdoses were confirmed due to NSO‐contaminated stimulants.

**Implications:**

Australia has strong monitoring capacity for NSOs post‐consumption. Harms occurring among opioid‐naïve people unknowingly purchasing NSOs suggest a role for drug checking and cautious use of nitazene testing strips. Expanding take‐home naloxone availability in entertainment settings, and to all who use drugs, is warranted.

**Conclusion:**

This review demonstrated the recent and limited emergence of NSOs in Australia. Demand for NSOs is not yet clear; availability and harms are rapidly changing.

## 1 Introduction

In recent years, the proliferation of novel synthetic opioids (NSO) has emerged as a global public health concern [1, 2]. Specifically, the opioid crisis in the US, now driven by synthetic opioids, has resulted in reduced life expectancy and overwhelmed healthcare systems [3]. Approximately 75,000 drug‐related deaths in the United States between April 2020 and April 2021 were attributed to synthetic opioids [2], a notable increase from 2015, when the US Centre for Disease Control reported 10,000 deaths due to synthetic opioids, largely driven by fentanyl analogues [4]. Similarly, in Canada, opioid‐related deaths predominantly involve fentanyl or NSOs [2].

Opioids fall into three broad categories: naturally‐derived (extracted from opium poppy plants), semi‐synthetic (such as oxycodone, manufactured by modifying an alkaloid found in opium) and synthetic such as fentanyl, entirely manufactured in laboratories [2]. Fentanyl, a potent synthetic opioid, was first developed in the late 1950s and became favoured for medical purposes due to its higher effectiveness as an analgesic compared to existing options at the time, such as morphine [2]. Fentanyl analogues (e.g., furanylfentanyl and ocfentanil) and other NSOs (e.g., protonitazene and U‐47700) have since emerged as higher potency synthetic opioids [2]. The exploration of nitazenes for medical use in the 1950s by pharmaceutical companies was abandoned due to safety concerns, including a high risk of overdose [5]. For the purposes of this review, we focus on NSO, defined as emerging novel psychoactive opioid substances that were not under international control at the time they emerged on the market.

According to the European Drug Report released in 2024 [6] (results to the end of 2023), 81 novel opioids had been identified since 2009. Notably, six of the seven novel opioids identified in 2023 were nitazenes. Nitazenes are often highly potent, but due to the cessation of research on these substances in the 1950s, little is known about their effects. Etonitazene, for example, is estimated to be 10 times more potent than fentanyl and 500 times more potent than heroin [7].

Over the past 5 years, since isonitazene was first detected in 2019 [8], nitazenes have been emerging in illicit drug markets in various parts of the world, linked to a growing number of drug‐related deaths and poisonings [9, 10]. In the United Kingdom, a report drawing on data from the National Crime Agency stated that over 170 deaths have been linked to nitazenes in the 12 months preceding May 2024 [11], representing approximately 7% of all opioid‐related deaths (*n* = 2551) in the United Kingdom in 2023 [12].

Australia has recorded steady increases in pharmaceutical opioid‐ [13] and heroin‐related harms over the last two decades [14] reflecting continued opioid use in this country. Indeed, more than 1.9 million people in Australia initiated pharmaceutical opioids every year from 2013 to 2017, though opioid prescribing, and particularly high‐dose opioid prescribing, has been decreasing in recent years [15]. Recent changes in Australian opioid markets, including tighter restrictions on pharmaceutical opioid prescribing and disruptions to heroin supply due to the COVID‐19 pandemic [16], have the potential to create a market for novel synthetic opioids. The North American experience with fentanyl‐related deaths underscores the urgency of addressing the emergence of novel synthetic opioids in Australia. While there is growing evidence on the global impact of novel synthetic opioids, particularly in regions like North America and Europe, evidence of the impact of these substances in Australian markets is limited. Due to the relative recency and speed of their emergence locally, there remains a gap in the literature for a synthesis of evidence surrounding NSO prevalence, patterns of use, and related harms in Australia. This supports the need for a focused review to better understand the unique challenges posed by NSOs in the Australian context, where current evidence is still fragmented. Therefore, the aim of this scoping review was to determine what is known about NSOs and their emergence in Australian drug markets.

## 2 Methods

### 2.1 Form of Review

We chose to conduct a scoping rather than a systematic review to identify and map available evidence on NSOs in Australia [17]. We use the Preferred Reporting Items for Systematic Reviews and Meta‐Analyses (PRISMA) Scoping Review checklist to guide our methodology [18].

### 2.2 Research Question

Our primary research question for the scoping review is ‘What do we know about novel synthetic opioids in Australia?’ A secondary question we aimed to answer is ‘What do these findings mean for harm reduction responses in Australia?’

### 2.3 Search Strategy

The comprehensive search strategies used aimed to identify all relevant literature. Specifically, the scoping review aimed to capture the history of what is known about NSOs in Australia, including their prevalence in drug markets, to inform potential future impacts and responses required [19]. The databases PubMed/MEDLINE and Embase were used to search the academic literature from the year 2000 to February 2024, and updated in May 2024. Keywords used in these searches included: ‘novel synthetic opioids (NSOs)’, ‘novel psychoactive substances (NPS)’, ‘Australia’, and over 60 specific NSO and fentanyl analogue (e.g., ‘carfentanil’, ‘etonitazene’) terms (see Table A1 for details). Terms for fentanyl analogues were identified from previous papers [5, 20, 21, 22]. Grey literature searches were also conducted in February 2024, May 2024 and November 2024 using Google, searching for the terms: (i) ‘novel synthetic opioids’ and ‘Australia’; (ii) ‘fentanyl analogues’ and ‘Australia’; and (iii) ‘nitazenes’ and ‘Australia’. The first 100 searches that were returned were scanned for relevance and included in the paper if they had not been covered by the academic literature. The updated grey literature search in November 2024 yielded two additional academic references not captured in earlier PubMed/MEDLINE/Embase searches. Finally, more targeted searches for government reports from various drug monitoring systems (e.g., the Prescription Recreation and Illicit Substance Evaluation (PRISE) and Western Australian Illicit Substance Evaluation (WISE) programs), reports on NSO seizures, and searches for public health/drug alerts in all states/jurisdictions in Australia were conducted and scanned for alerts on NSOs. This also included drug notifications published by CanTEST, Australia's first fixed site drug checking service in the Australian Capital Territory (ACT) and by CheQPoint, the drug checking service in operation in Queensland. Grey literature searches were filtered to provide results only from 2013—November 2024 as there were no mentions in the academic literature prior to 2013.

### 2.4 Inclusion Criteria

Studies included in this review were required to focus specifically on NSOs in Australia. Articles were included if they provided information on the consumption patterns, prevalence rates, hospital presentations, toxicology reports, harms and seizures related to NSOs in Australia. Studies were excluded if there was no information related to NSOs or if they did not contain data related to Australia.

### 2.5 Screening Process

Using Covidence, a web‐based program facilitating article screening and data extraction for academic reviews [23], titles and abstracts of retrieved articles were screened independently by two reviewers to identify potentially relevant studies. Full‐text articles were then assessed by two reviewers for eligibility based on the inclusion criteria outlined above. Any conflicts in this process were resolved through discussion and consensus between the reviewers.

## 3 Results

**FIGURE 1:**
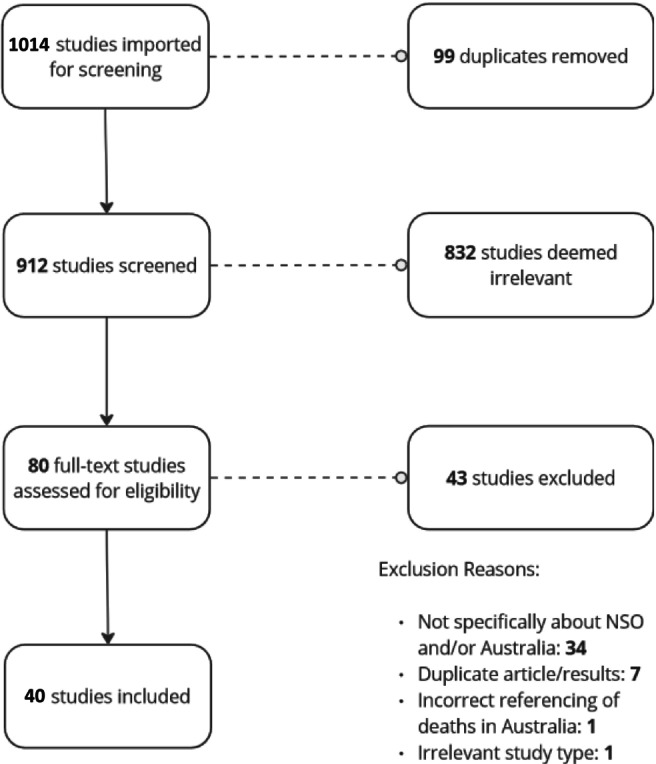
PRISMA diagram.

A total of 1014 peer‐reviewed studies were imported for screening, with 99 of these being identified as duplicates. After duplicates were removed, 912 imported articles went through title and abstract screening, with 832 deemed not relevant. This left 83 articles for full‐text review. Of these, 43 were excluded, leaving 40 articles for inclusion. A summary of the studies identified and reasons for exclusion is provided in the PRISMA diagram (Figure 1).

### 3.1 Peer‐Reviewed Publications

We group and present the results of our searches by the methodology of the studies (Table 1).

**TABLE 1:** Study characteristics and summary findings from peer‐reviewed publications.

	Author, year and title	Methodology	Main findings
1	Bade et al. (2020): Determination of 21 synthetic cathinones, phenethylamines, amphetamines and opioids in influent wastewater using liquid chromatography coupled to tandem mass spectrometry	Monitoring Wastewater	5 NSOs screened for in wastewater samples 19 Dec 2018‐1 Jan 2019, 3 NSOs detected (butyryl fentanyl, furanyl fentanyl and valeryl fentanyl).
2	Bade et al. (2019): Investigating the appearance of new psychoactive substances in South Australia using wastewater and forensic data	Monitoring Wastewater/toxicology samples	3 NSO detections: P‐Flourobutrylfentanyl detected Aug 2015, U‐47700 in Feb 2016, Ocfentanil in June 2016.
3	Bade et al. (2019): LC‐HRMS suspect screening to show spatial patterns of New Psychoactive Substances use in Australia	Monitoring Wastewater	U‐4700 detected but not confirmed in wastewater WA.
4	Bade et al. (2020): What is the drug of choice of young festival goers?	Monitoring Wastewater	NPS (including opioids) screened for. Only norfentanyl (most likely from fentanyl use) detected.
5	Bade et al. (2024): Early identification of the use of potent benzylbenzimidazoles (nitazenes) through wastewater analysis – two years of data over 22 countries	Monitoring Wastewater	Four unique samples of protonitazene detected across two sites 2023/24. Etonitazepyne detected at one site 2023/24.
6	Barratt et al. (2018): Urine drug screening for early detection of unwitting use of fentanyl and its analogues among people who inject heroin in Sydney, Australia	Monitoring Urine drug screening at medically supervised injecting centre, Sydney	No cases detected.
7	Barratt et al. (2024): Adulteration and substitution of drugs purchased in Australia from cryptomarkets: An analysis of Test4Pay	Monitoring Instances of drug adulteration in Australian purchases from cryptomarkets	Of 103 samples, no fentanyl contamination detected. 1 purchase (advertised as) metonitazene did not contain the advertised substance. although the substance resembled metonitazene and was likely a nitazene not in the testing library.
8	Black et al. (2020): Toxicological analysis of serious drug‐related harm among electronic dance music festival attendees in New South Wales, Australia: A consecutive case series	Monitoring Toxicology samples from ambulance/emergency department drug toxicity presentations following attendance at music festivals	Forty cases. One case of fentanyl detection but NOT fentanyl analogue. No other NSO detected.
9	Boumba et al. (2017): The analysis of 132 novel psychoactive substances in human hair using a single step extraction by tandem LC/MS	Monitoring Hair analysis from 23 medico‐legal cases where NPS suspected to be involved or found in blood	Acetyl fentanyl found in 2 cases.
10	Brett et al. (2022): Wastewater analysis for novel psychoactive substances at music festivals across New South Wales, Australia in 2019–2020	Monitoring Analysis of wastewater from six music festivals	Traces of norfentanyl detected at one festival (likely to reflect pharmaceutical fentanyl use).
11	Darke et al. (2022): Characteristics of fatal ‘novel’ synthetic opioid toxicity in Australia	Mortality Review of all deaths involving NSOs (2000–2021) from national coronial information system	31 cases, 10 NSOs identified. 21 cases where fentanyl analogues identified (predominantly furanylfentanyl/acetylfentanyl), and 12 cases of non‐fentanyl analogues identified, predominantly U‐47700.
12	Curtis et al. (2024): Identification of the novel synthetic opioid *N*‐pyrrolidino isotonitazene at an Australian drug checking service	Monitoring Analysis from sample at CanTEST drug checking service	N‐pyrrolidino isotonitazene was detected in September 2024 in an expected oxycodone sample.
13	Darke et al. (2024): Emergence of deaths due to nitazene toxicity in Australia	Mortality Examination of nitazene‐related deaths in Australia	17 deaths, all deemed unintentional.
14	Di Rago et al. (2022): Toxicity of heroin laced with synthetic opioidβ ‐U10 and etizolam	Monitoring Hospital presentations for opioid poisonings as part of EDNAV monitoring system.	7 cases where β‐U10 detected.
15	Drummer (2019): Fatalities caused by novel opioids: a review	Mortality International review of NSO related deaths	No Australian cases published at the time the review was conducted. Deaths primarily occurring in the US and Western Europe.
16	Greene et al. (2023): Characteristics of analytically confirmed novel opioid exposures in patients presenting to emergency departments within the state of Victoria, Australia	Monitoring Toxicology samples from people presenting to emergency department (EDNAV).	Illicit drug presentations 8/363 (< 1%) of cases detected NSOs. Seven of these were nitazene compounds.
17	Harris et al. (2024): Bedside urine testing for fentanyl in self‐reported heroin users in a tertiary Brisbane emergency department	Monitoring Brisbane toxicology unit analysis for urine samples testing the presence of fentanyl and fentanyl analogues in cases involving self‐reported heroin use	No detection of fentanyl/fentanyl analogues.
18	Jaunay et al. (2024): Monitoring the use of novel psychoactive substances in Australia by wastewater‐based epidemiology	Monitoring Wastewater – national samples analysed for NPS	Traces of valeryl fentanyl found in Apr 2022 sample in 3 regional sites.
19	Lam et al. (2022): Infrequent detection of unintentional fentanyl use via urinalysis among people who regularly inject opioids in Sydney and Melbourne, Australia	Monitoring Urine analysis from supervised injection facilities sample to identify unintentional fentanyl or fentanyl analogue consumption	2% of 911 participants reported intentional pharmaceutical fentanyl use (predominantly fentanyl patches). 2 cases were also found of unintentional fentanyl use. No fentanyl analogues were detected.
20	Lamy et al. (2020): Listed for sale: Analysing data on fentanyl, fentanyl analogs and other novel synthetic opioids on one cryptomarket	Monitoring Analysis of cryptomarket online listings for sale of fentanyl analogues and other NSOs	395 listings for non‐pharmaceutical fentanyl, 38.3% of these listings with Australia as country of origin. 224 listings for NSO U‐48,800, 3.1% with Australia as country of origin.
21	Lamy et al. (2021): ‘Etazene, safer than heroin and fentanyl’: Non‐fentanyl novel synthetic opioid listings on one darknet market	Monitoring Analysis of online cryptomarket listings for sale of non‐fentanyl NSOs	NSO 2‐MAP‐237 listed: 52.4% of sales where country of origin and intended destination was Australia. 10.3% of sales where intended destination was Australia.
22	Maplesden et al. (2023): An intoxication involving 2‐methyl AP‐237 and AP‐238 from Victoria, Australia: Case report	Monitoring Emergency department presentation toxicology analysis (EDNAV)	NSOs (2‐methyl AP‐237 and AP‐238) identified.
23	Marlin et al. (2017): The characterisation of carfentanil sales on a major darknet cryptomarket	Monitoring Analysis of cryptomarket listings of carfentanil for sale	Evidence of carfentanil listings for sale in Australia.
24	McCutcheon et al. (2019): An early warning system for emerging drugs of concern in the emergency department: Protocol for the Western Australian Illicit Substance Evaluation (WISE) study	Monitoring Protocol for emergency department monitoring system (WISE)	Protocol only, no findings.
25	Moss et al. (2019): An acetyl fentanyl death in Western Australia	Mortality Death case report/toxicology analysis	Fatality involving acetyl fentanyl.
26	Nash et al. (2019): A Fatality Involving Furanylfentanyl and MMMP, with Presumptive Identification of Three MMMP Metabolites in Urine	Mortality Death case report/toxicology analysis	Fatality involving furanylfentanyl.
27	Nielsen et al. (2023): Monitoring for fentanyl within Australian supervised injecting facilities: Findings from feasibility testing of novel methods and collaborative workshops	Monitoring Analysis of injecting equipment at supervised injecting facilities	Replication of Lam (2022) results for urine samples, however, analysed injecting equipment from 59 overdoses returned no detection of NSOs.
28	Partridge et al. (2018): A Case Study Involving U‐47700, Diclazepam and Flubromazepam‐Application of Retrospective Analysis of HRMS Data	Mortality Death case report/toxicology analysis	Fatality involving U‐47700 & AH‐7921.
29	Partridge et al. (2024): A cluster of multi‐drug intoxications involving xylazine, benzimidazole opioids (nitazenes) and novel benzodiazepines in South Australia	Monitoring Analysis from emergency department presentations (EDNA)	Three cases of nitazene‐related (protonitazene and metonitazene) emergency department presentations.
30	Rauf et al. (2021): Causes, Nature and Toxicology of Fentanyl‐Analogues Associated Fatalities: A Systematic Review of Case Reports and Case Series	Mortality International review of fentanyl‐analogue deaths	Majority of published literature on fatalities from the US (96.8%), followed by the UK (3%). Only one case from Australia at this point (as reported by Moss et al. 2019).
31	Roxburgh & Nielsen (2022): Twenty‐year trends in pharmaceutical fentanyl and illicit fentanyl deaths, Australia 2001–2021	Mortality Study of fentanyl deaths (2001–2020) and the proportion attributable to illicitly manufactured fentanyl and fentanyl analogues in Australia	A small proportion of deaths attributed (22 of 833) to fentanyl analogues.
32	Schumann et al. (2023): Intoxications in an Australian Emergency Department Involving ‘Nitazene’ Benzylbenzimidazole Synthetic Opioids (Etodesnitazene, Butonitazene and Protonitazene)	Monitoring Emergency department presentations toxicology analysis (EDNAV)	The first detection of nitazene toxicity cases in Australia. 2 cases – etodisnitazene, protonitazene and butonitazene detected.
33	Smith et al. (2022): The Emerging Drugs Network of Australia: A toxicosurveillance system of illicit and emerging drugs in the emergency department	Monitoring Description of toxicosurveillance system for illicit substances (EDNA)	Protocol only, no findings.
34	Syrjanen et al. (2023): The Emerging Drugs Network of Australia—Victoria Clinical Registry: A state‐wide illicit substance surveillance and alert network	Monitoring Description of illicit substances surveillance system (EDNAV)	Protocol only, no findings.
35	Syrjanen et al. (2023): Characterisation of illicit drug exposures within an early warning system: medical record documentation performs poorly compared to comprehensive toxicological analysis of biofluid	Monitoring Analysing emergency department presentations for illicit drug exposure (EDNAV)	Out of 1088 suspected illicit drug exposures, eight analytically confirmed cases of novel opioid exposure.
36	Syrjanen et al. (2023): From signal to alert: A cluster of exposures to counterfeit alprazolam tablets containing five novel benzodiazepines	Monitoring Article outlining how EDNAV works to provide drug alerts using case example	Illicit opioids (unnamed) co‐detected in four cases.
37	Syrjanen et al. (2023): A risk‐based approach to community illicit drug toxicosurveillance: operationalisation of the Emerging Drugs Network of Australia‐ Victoria (EDNAV) project	Monitoring Outlining examples of EDNAV drug detection	Among 8 samples, 1 case of ‘novel opioid adulterated heroin’, and 1 case of protonitazene sold as ketamine (Likely the source for VIC 2022 drug alert below).
38	West et al. (2021): Early Warning System for Illicit Drug Use at Large Public Events: Trace Residue Analysis of Discarded Drug Packaging Samples	Monitoring Detecting Illicit drugs in discarded drug packaging	Screened for fentanyl and fentanyl analogues but none detected.
39	West et al. (2022): Trace residue identification, characterisation, and longitudinal monitoring of the novel synthetic opioid β‐U10, from discarded drug paraphernalia	Monitoring Analysis of discarded drug paraphernalia	β‐U10 detected in at least 2 samples from discarded drug paraphernalia.
40	White et al. (2023): Acute metonitazene poisoning reversed by naloxone	Monitoring Emergency department/hospital presentation case report of NSO intoxication	Metonitazene identified in an individual who had purchased what they thought was ‘spray oxycontin’ online.

Abbreviations: EDNA, Emerging Drugs Network of Australia; EDNAV, Emerging Drugs Network of Australia – Victoria; HRMS, high resolution mass spectrometry; LC‐HRMS, liquid chromatography high‐resolution mass spectrometry; LC/MS, liquid chromatography mass spectrometry; MMMP, 2‐methyl‐4′‐(methylthio)‐2‐morpholinopropiophenone; NPS, novel psychoactive substance; NSO, novel synthetic opioid; WISE, Western Australian Illicit Substance Evaluation.

### 3.2 Monitoring Systems

Thirty articles [24, 25, 26, 27, 28, 29, 30, 31, 32, 33, 34, 35, 36, 37, 38, 39, 40, 41, 42, 43, 44, 45, 46, 47, 48, 49, 50, 51, 52, 53, 54, 55] focused on monitoring systems, seven of which utilised wastewater analyses as a means of screening for NSO use in the population. Five articles gathered samples from wastewater treatment plants, ranging from five‐year collection periods in Australia [26], two‐year collection periods across 22 countries including Australia [54], to one‐time cross‐sectional collection at events in Australia [24, 25]. These articles covered the period June 2012 to January 2024, and in that time, there were 15 unique detections of NSOs (including butyryl fentanyl, fluorobutryl fentanyl, furanyl fentanyl, ocfentanil, valeryl fentanyl, U‐47700, protonitazene and etonitazepyne); the earliest detection (flourobutryl fentanyl) occurred in 2015. A more targeted wastewater monitoring study was reported by Brett and colleagues (2022), with samples collected from portable toilets at six different music festivals in New South Wales over a 12‐month period (March 2019–March 2020) [32]. Norfentanyl was detected at one festival, likely indicating the use of fentanyl. There were no NSO detections reported in this study.

Eight articles [34, 35, 41, 45, 47, 48, 50, 56] reported findings from the Emerging Drugs Network of Australia (EDNA) monitoring system (seven from the Victorian arm [EDNAV] of the system and one from South Australia), analysing toxicology samples from emergency department presentations in cases of suspected drug poisoning. These articles analysed data captured between October 2020 and December 2023. There were 30 unique NSO‐related cases reported across seven articles, including the first detection of nitazenes in Australia in 2021 [45]. Greene and colleagues [35] found eight (2.2%) of 363 cases of opioid detections in emergency department presentations involving NSOs; one detection of β‐U10 and one detection of AP‐237, with the remainder being nitazene compounds (protonitazene *N* = 5, etodesnitazene *N* = 1 and butonitazene *N* = 1). One article presented three cases of nitazene‐related (protonitazene and metonitazene) emergency department presentations in South Australia in December 2023 [56] also involving the first reports of bromazolam and nitrazolam in Australia. Three articles [43, 46, 49] were protocols outlining the nationally capability of NSO monitoring and the EDNA system. Three additional articles (unrelated to EDNA) reported on drug poisoning cases [30, 36, 53]. In one case, metonitazene was detected in a product purchased as ‘oxycontin spray’ [53].

Five articles [28, 38, 44, 51, 52] reported on the analysis of injecting equipment and urinalysis at the two supervised injecting facilities in Australia. These articles analysed data captured between 2017 and 2021. Lam and colleagues [38] report on a sample of 911 participants recruited from supervised injecting facilities in Sydney and Melbourne. Although there were reports of both intentional (*n* = 18) and unintentional (*n* = 2) fentanyl use, there were no cases of NSO/fentanyl analogue detection despite testing for a range of fentanyl analogues. Further, Nielsen and colleagues [44] tested injecting equipment from 59 opioid overdoses in a medically supervised injecting facility, with no detection of NSOs. West and colleagues [52] reported there were at least two cases of the NSO β‐U10 being detected in discarded drug paraphernalia at the Melbourne supervised injecting facility. One additional article, focused on hair analysis, reported two detections of acetylfentanyl [31].

Four articles, analysing data captured between March 2018 to August 2023 [29, 39, 40, 42] reported cryptomarket monitoring of online sales of NSOs and online drug checking services. Of these, all reported evidence of the intended sale of NSOs being shipped to or from Australia. Lamy and colleagues [39] reported findings of listings for 11 fentanyl analogues and 17 other NSOs. Among these substances, there were 224 listings for U‐48800, with 3.1% listing Australia as the country of origin in these listings. In a subsequent study, Lamy and colleagues [40] identified 883 listings relating to 17 non‐fentanyl NSOs (representing 2.9% of all opioid listings). Of 53 listings for U‐47700, 88% had Australia listed as the country of origin; of 136 listings for 2‐AP‐237, over half (52.4%) had Australia listed as the country of origin and country of destination. In an analysis of data extracted from Test4Pay, a darknet forum reporting results of analytic testing conducted by a drug testing service in Canada, Barratt and colleagues [29] found that of 103 samples that were purchased in Australia, only one sample contained evidence of metonitazene likely being present. The authors noted the exact chemical structure was not present in the testing library.

Finally, one article reported on findings from CanTEST, where N‐pyrrolidino isotonitazene was detected in September 2024 in an expected oxycodone sample [55].

### 3.3 Mortality Studies

Eight articles [4, 20, 21, 57, 58, 59, 60, 61], analysing data from 2001 to 2023, reported on NSO‐related deaths in Australia. Three were single or cluster event case studies (providing details of the toxicology analyses), while the other five were broader reviews on NSO‐related deaths with results spanning over extended time periods. The methodology for the reviews varied, with three Australian‐focused studies [20, 21, 61] utilising data from the National Coronial Information System, while the remaining two studies [4, 60] conducted reviews of international academic literature reporting on NSO‐related fatalities. Overall, approximately 51 unique Australian cases of NSO‐related deaths were reported [20, 21, 61]. Of 22 deaths related to fentanyl analogues, acetylfentanyl, furanylfentanyl and flourofentanyl variants were most commonly detected. There were also 12 deaths in which other NSOs were identified in toxicology—predominantly U‐47700—occurring from 2016 onwards [20]. There were reportedly 17 deaths attributable to nitazene compounds, occurring from 2021 onwards Australia wide [61]; however, this is expected to be a significant underestimate as many of these cases may still be under investigation; twenty‐nine confirmed deaths in total have been reported to date in the media and by the Coroner's Court of Victoria (see the Grey Literature Mortality section below). In addition, as of November 2024, there are now 22 confirmed nitazene‐related deaths that have occurred in Victoria since 2021 [62].

**FIGURE 2:**
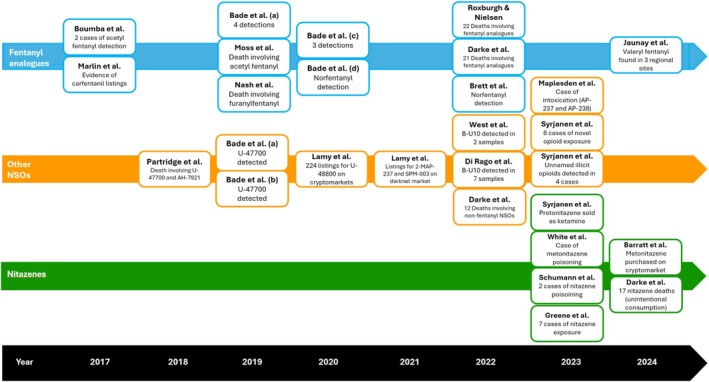
Timeline of academic literature reporting novel synthetic opioids (NSO) in Australia.

Figure 2 is a visual timeline of articles reporting on NSOs in Australia.

### 3.4 Grey Literature

#### 3.4.1 Police Seizures

Grey literature searches returned reports of more than 50 NSO seizures in Australia. In January 2018, carfentanil was detected in a seizure in the ACT [63] (Table 2). In August 2023, an individual was charged by authorities for allegedly attempting to import 97 nitazene tablets (among various other drugs) into Australia to sell online [64]. In October 2023, police charged a NT individual for importing 5 g of metonitazene from the UK [64], and in the same month 742 tablets containing metonitazene were seized coming into Australia by the Australian Border Force [64]. The Australian Federal Police reported a total of 47 nitazene seizures detected at the border between July 2023 and October 2024, and eight nitazene seizures in Queensland during this time [65]. Finally, in July 2024, two people were arrested following 198 g of nitazenes being seized at a property in South Australia [66].

**TABLE 2:** Summary findings of grey literature.

Author, year and title	Category/methodology	Main findings
Australian Federal Police (AFP) social media reporting on total nitazene seizures (Oct 2024)	Media release	47 nitazene border detections since July 2023 and 12 seizures in Queensland.
ACT Health (2018): Drug alert	Public drug alert/monitoring	Carfentanil detected in seizure.
ACT [CanTEST] (2022–2024): Drug Notifications	Public drug alert/monitoring	Three warnings about nitazenes.
AFP (2024): Rising imports of potent drug nitazene raises concern	Media release	5 g of metonitazene seized after being imported from the UK. Failed importation of 97 nitazene tablets, NSW man charged. 742 tablets being seized by the Australian Border Force, which contained metonitazene.
Australian Criminal Intelligence Commission (ACIC) 2019 National wastewater drug monitoring program report	Report of wastewater findings/monitoring	Fentanyl usage reported on but no mention of NSOs.
Broadhurst et al. (2020): Fentanyl availability on darknet markets	Report on cryptomarket availability of fentanyl/fentanyl analogues/monitoring	Highlights the availability of fentanyl/fentanyl analogues on darknet markets. Emphasises the affordability of these drugs and the fact that Australians are active on darknet markets could facilitate an increase in use of NSOs in Australia.
Bruno et al. (2023): New psychoactive substance markets and monitoring in Australia: An update	Summary of trends of NPS in Australia/monitoring	5 alerts for NSOs being detected but sold as other drugs (e.g., Acetylfentanyl sold as heroin).
Cooper 2024: Synthetic opioids ‘one of the biggest threats’ to drug‐related harm	Media report	Highlights the danger/threat of NSOs emerging in Australia based on the opioid crisis in North America.
Gorringe et al. (2023): Nitazenes nitty‐gritty: What are nitazenes? And why are they being found in heroin and ketamine in Australia?	Media report	General mention of nitazenes being detected in Australia.
Nielsen & Roxburgh & Armour: Opioids more potent than fentanyl have been detected in Australia. So what are nitazenes?	Web article	Article explaining nitazenes and the possible risks for Australia.
NSW Health (2019–2024)	Public drug alert/monitoring	5 warnings about acetylfentanyl, 4 warnings for nitazenes.
NSW (2024): NUAA: Drug Alerts: https://nuaa.org.au/drug‐alerts	Public drug alert/monitoring	1 warning for NSOs: Nitazenes have been found in multiple substances linked to opioid overdoses in NSW.
Northern Territory (2023): Association of Alcohol and Other Drug Agencies NT (AADANT) drug alert	Public drug alert/monitoring	1 Metonitazene seizure (small amount).
Prescription, Recreational and Illicit Substance Evaluation (PRISE) report 2018–2021	Report on the PRISE system/monitoring	New psychoactive substances were detected in 15% of the 390 tested cases. Acetylfentanyl the most commonly detected NPS. Carfentanil, flouro furanyl fentanyl, and etodesnitazene were among the other NSOs found.
Queensland (2023): Queensland Health: Alert issued over dangerous counterfeit tablets	Public drug alert/monitoring	Protonitazene found in tablets branded as other drugs (e.g., Xanax).
South Australia (2023): Aboriginal Health Council of South Australia: Protonitazene cases in South Australia	Public drug alert/monitoring	2 cases of protonitazene overdose.
Schumann et al. (2022): Intoxications in an Australian emergency department involving ‘Nitazene’ Benzylbenzimidazole Synthetic Opioids (Etodesnitazene, Butonitazene and Protonitazene)	Case report/monitoring	Report of 2 cases where NSOs detected: First case was a detection of etodesnitazene. Second case was a detection of protonitazene that also contained butonitazene.
Synthetic opioid detected in four found dead in Broadmeadows (2024)	Web article	Article reporting on case where four people were found dead in a Victorian residence after suspected nitazene overdose.
Department of Health, Victoria (2022–2024)	Public drug alert/monitoring	4 warnings for nitazenes being sold as other drugs e.g., cocaine.

Abbreviations: ACT, Australian Capital Territory; NSO, novel synthetic opioid; NSW, New South Wales.

#### 3.4.2 Health Department Issued Drug Alerts

Since 2019, 51 total public drug alerts were found across all Australian states and territories [67, 68, 69, 70, 71, 72]. A third (*n* = 18) of these public drug warnings were for NSOs, the earliest occurring in 2020, with five occurring in 2023 and four reported up until September 2024. Of the NSO warnings, 12 were related to nitazenes (predominantly protonitazene) being detected in and sold as other drugs in different states/territories (five from New South Wales, four from Victoria, two from South Australia, and one from Northern Territory and Queensland). Earlier warnings from 2020 and 2021 typically related to acetylfentanyl being found as contaminants in heroin, cocaine, and methamphetamine. However, more recent public drug alerts from 2023 and 2024, warn about nitazenes being found as contaminants in (notably non‐opioid) drugs such as MDMA, ketamine, 3C‐P and Xanax. Nitazenes were detected in drug samples related to 20 overdoses occurring in NSW in April 2024 [73] and another 4 overdoses in May 2024 [74]. CanTEST, Australia's first fixed site drug checking service, has released 24 drug notifications since September 2022 [75]. Of these, three involved nitazenes; metonitazene in December 2022, protonitazene in May 2024, and isotonitazene in September 2024. The traces of metonitazene and isotonitazene were both found in unmarked yellow pills, initially presumed to be counterfeit oxycodone, while the protonitazene was detected in a sample of ‘brown powder’. CheQpoint, the Queensland drug checking service, has released five drug notifications since they commenced operations in April 2024 [76], one of which related to the presence of protonitazene detected in a sample of counterfeit oxycodone tablets [77].

#### 3.4.3 Mortality Reports

In March 2024, the Coroner's Court of Victoria released a report stating there have been at least 16 deaths involving nitazenes occurring in Victoria since the beginning of 2021 [78]. The report documented a specific case in which the deceased likely believed they were using heroin but unknowingly consumed metonitazene instead. In June 2024, police reported four additional overdose deaths linked to nitazenes (later confirmed to be protonitazene contaminated cocaine) at a residence in Victoria [79], and July 2024 police reporting confirmed seven deaths and 13 known non‐fatal overdoses in South Australia since January 2022 related to nitazenes [66]. Two nitazene‐related overdoses and two nitazene deaths were reported in NSW in Sept 2024 after individuals consumed what they thought was cocaine [80].

[Correction added on 26 July 2025, after first online publication: This media article (80) incorrectly referenced a NSW Health drug alert as being related to nitazenes. The drug alert was in fact for heroin. Therefore the above mentions of nitazene‐related overdoses and nitazene deaths in NSW were actually due to heroin.].

#### 3.4.4 Monitoring Systems

Grey literature reports included the Prescription, Recreational, Illicit Substance Evaluation (PRISE), a summary of toxicology reports from drug intoxications in New South Wales [81]. These data are gathered from cases attended to by various public health rapid response services, including cases of intoxication at music festivals and emergency department presentations. Fentanyl analogues and nitazenes (in a much lower proportion) were detected in approximately 30 of the 390 (7.7%) tested cases. Acetylfentanyl was most commonly detected, while carfentanil, flouro furanyl fentanyl, and etodesnitazene were among the other NSOs detected. Urinalysis monitoring of detainees in prisons, conducted by the Australian Institute of Criminology, identified fentanyl analogue β‐hydroxyfentanyl in (0.5%) of the detainees sampled (*n* = 13) in July of 2019 [82]. A briefing report on nitazenes from the National Centre for Clinical Research on Emerging Drugs identified 22 nitazene cases (protonitazene, metonitazene, isotonitazene and etonitazepyne) in Queensland, South Australia, Victoria and Western Australia [83]. The National Drug and Alcohol Research Centre reported on the availability of nitazenes for purchase on cryptomarkets from February 2023 to January 2024 [84]. A total of 1751 listings for nitazenes were identified; 75% of these listings were advertised as deliverable to Australia [84]. Nitazene listings comprised less than 1% of all cryptomarket drug listings during the period [84].

## 4 Discussion

To date, there have been 40 published articles reporting on the monitoring and detection of NSOs in Australia. The majority of these (30 articles) were based on data from monitoring systems in which 49 unique NSO detections (excluding cryptomarkets) were reported. Notably, earlier reports relate to fentanyl analogues and U‐47700, while the more recent reports relate to nitazenes, with the first Australian nitazene detection reported in 2021 [45] and eight nitazene detections reported in ED settings in 2023 [35].

A similar trend was seen in the mortality studies reporting on NSO‐related deaths. Fentanyl analogues made up the largest proportion of these deaths from 2013 onwards, with nitazene deaths increasingly being reported from 2021 [61]. The two sources for nitazene related deaths in this review (academic and grey literature), reported conflicting results, with the academic paper [61] likely being an underestimate, due to some of the more recent coronial investigations remaining open and therefore not finalised. This highlights a major limitation for researchers outside the coronial system in capturing up‐to‐date data, due to the lack of access to cases which are still under investigation. Coronial investigations can be lengthy, making it difficult to report on mortality figures in a timely way.

In documenting trends relating to NSO use in Australia, there is an important distinction between intentional and unintentional use. Some of the EDNAV publications were able to report whether NSO consumption was intentional, but few other studies have been able to ascertain this. Understanding the demand (i.e., intentional purchase and consumption) for NSOs in Australia is a gap in our knowledge, which, if addressed, would be important to inform harm reduction responses. Early findings from CanTEST suggest the detections of nitazenes were unexpected substances (i.e., purchase of nitazenes was unintentional) [85], while the CheQpoint warning of nitazenes detected in counterfeit oxycodone [77] is indicative of an unintentional purchase. Findings demonstrate the value of these services.

Other results further highlight challenges with monitoring the rapidly evolving range of nitazenes in circulation. For example, one metonitazene‐like substance was detected, but was not able to be identified beyond being classed as a nitazene, as it was not matched to any substances within the study's testing library [29]. This highlights challenges where there is a lack of technical capability in detecting emerging opioids [4]. It may also highlight the differences in toxicology testing protocols across jurisdictions, where not all substances will be routinely screened for. If we are to accurately identify trends in mortality due to novel synthetic opioids, the implementation of routine opioid screening protocols at a national level would be useful.

Grey literature provided reports on NSO‐related seizures and public drug alerts. These sources were useful to capture more up‐to‐date instances of NSO market indicators not yet reported in academic literature. Specifically, the public drug alerts highlighted a changing trend in the last 2 years of nitazenes being increasingly detected as contaminants in illicit drugs, with fentanyl analogues more commonly detected prior to 2023. Notably, nitazenes have been found in stimulant drugs (including MDMA, cocaine and 3C‐P) [68], which have vastly different mechanisms of action and effects compared to opioids. This presents an increased risk of overdose for people who might be opioid naïve, and therefore not familiar with the potency of opioids or how opioids might affect them. The relatively recent emergence of NSOs in Australia and reports of nitazene‐related deaths highlight the potentially concerning pace at which nitazenes are impacting drug markets in Australia.

It is challenging to determine the specific factors driving the emergence of NSOs in Australian drug markets. One likely factor is the predicted dramatic reduction in the global heroin supply resulting from the opium ban (with a 95% reduction in opium cultivation recorded in Afghanistan) enforced by the Taliban in 2022 [86]. Scarcity of heroin is likely to increase prices for heroin. With synthetic opioids, which are more easily manufactured and distributed internationally (their stronger potency makes it possible to move heroin‐equivalent doses in much smaller shipments), shortfalls in the global illegal opioid supply can be overcome [2]. Furthermore, there is some evidence that some people are using NSO as a drug of choice, purchasing them online through cryptomarkets [87].

The other concerning emerging trend is the detection of NSO in counterfeit pharmaceutical opioid pills such as oxycodone [75]. This potentially creates a new at‐risk population of people who may be seeking pharmaceutical opioids outside of the medical system, for medical and non‐medical purposes, given the increasing restrictions on prescribing and supply of opioid analgesics in Australia.

The threat of unintentional NSO consumption underlines the need for better early warning systems, so the public can be alerted to specific cases of drug contamination. The Know Community [88] is attempting to address this need by collating public drug alerts from each state and territory of Australia, which were previously reported separately. While this will assist with providing a more synthesised and comprehensive drug warning system, to be effective, it relies on well‐organised collaborative information sharing across organisations and jurisdictions for the timely dissemination of warnings to the public, which are often delayed. Community‐based drug checking services are an important initiative to address the harms related to unintentional NSO consumption.

The potential for NSOs to cause serious harm supports upscaling of harm reduction responses. Take‐home naloxone has proven to be an effective tool for reversing synthetic opioid overdoses [89]. Because of the potency of NSOs, in some cases, it is often suggested that higher doses of naloxone have been required, although there is growing evidence that usual doses are effective [89]. Consideration of how take‐home naloxone programs may need to be adapted, including any changes to the administration of naloxone by emergency response services is an area for further work. It is critical that community messaging reinforces that naloxone is effective to reverse NSO overdoses, with misinformation about ‘fentanyl‐resistant naloxone’ likely to undermine the effectiveness of harm reduction programs. Extending naloxone availability to more recently identified at‐risk populations (such as people who use stimulants) may help to reduce NSO related harms. This includes opioid naïve groups, who could also benefit from education surrounding NSOs. Greater availability of take‐home naloxone in entertainment settings is also important given the detection of nitazenes as contaminants in non‐opioid drugs like MDMA and cocaine [68].

Our review highlights the valuable information about illicit drug markets being identified through drug checking services [90], especially given the emergence of NSOs/nitazenes in drugs sold as stimulants and ketamine [67, 68]. Drug testing strips also exist, and recent evidence indicates their efficacy in the detection of various nitazenes; however, recent research identifies that a range of nitazenes are not able to be detected by these instant strips [91]. The limitation in interpreting negative results with instant test strips, alongside common user error in their interpretation, suggests that fixed‐site drug checking services will remain essential in harm reduction efforts. There are several types of drug checking technologies, ranging from fast and portable options such as reagent tests and Fourier Transform Infrared Spectroscopy to slower fixed‐site options like Liquid Chromatography‐Mass Spectrometry [92, 93]. Faster portable options are typically more affordable but lack the sensitivity of more expensive lab‐based options [92, 93]. Therefore, drug‐checking methods and technologies used will depend on specific needs within different local contexts. Currently, only four jurisdictions in Australia have implemented drug checking services in some form (ACT, Queensland, Victoria and NSW [94, 95]), despite NSO‐related harms occurring across the country; nitazene detections have been reported in every Australian jurisdiction to date except for Tasmania. Expanding drug checking services, both fixed and event‐based, across all jurisdictions is an important strategy in preparedness for the broader emergence of nitazenes in Australian drug markets. It must be noted that these services need to be technically equipped to detect nitazenes in the low concentrations they are likely to occur [92, 93] and not only the more commonly used drug types.

One strength of this scoping review is the inclusion of published academic literature alongside grey literature, addressing limitations with the timeliness of published research through supplementing it with other sources. The limitation of publication lag‐times is particularly evident in the context of emerging substances like nitazenes, where trends may evolve more quickly than the literature can report on. Additionally, the availability of mortality data for NSO‐related deaths is clearly incomplete, in part due to the time taken to conclude coronial investigations, leading to an underestimation of the true extent of NSO‐related mortality. The variability of NSOs also presents another challenge in successful detections of these substances. Because of the high potency of some nitazenes, they may only be present in a low concentration within a given sample, meaning only the best instruments are able to detect them, and even then, concentrations can be below the limit of detection [4]. A limitation of the grey literature, such as media reports, is that they lack the rigorous peer‐review process of academic publications. While care has been taken to identify and account for duplicate data, it is possible that some sources have overlapped, such that the same signal or event may have been reported across different sources.

Our findings suggest that Australia has developed a relatively robust set of monitoring systems to enable detection of NSO and related harms. The challenge is to do so in as close to real‐time as possible to minimise the impacts of the appearance of NSO in local drug markets. Drug checking services and public health alerts are a vital part of real‐time monitoring; however, differing data privacy legislation across jurisdictions often impedes timely sharing of important information. One of the gaps in our understanding in relation to NSO in Australia is related to our understanding of the extent to which people are intentionally seeking these substances out for consumption versus unintentional consumption.

## 5 Conclusion

The detection and emergence of NSOs, particularly nitazenes, in Australia presents a growing public health concern. To address the ongoing risks posed by NSOs, particularly through unintentional consumption, enhanced harm reduction measures such as the expansion of drug checking services, cautious use and interpretation of nitazene testing strips, and expanded availability of take‐home naloxone are critical. Further, enhanced early warning systems, with timely reporting of drug alerts, are essential for timely public dissemination of drug contamination risks. These initiatives, combined with increased knowledge among the broad range of populations who may be affected by NSO overdoses, will be key in preventing and responding to harms associated with NSOs in Australia.

## Author Contributions

**Emmanuel Mammoliti:** Writing – original draft (lead), formal analysis (lead). **Suzanne Nielsen:** Methodology (equal lead), writing – reviewing and editing. **Amanda Roxburgh:** Conceptualisation (lead), methodology (equal lead), formal analysis (supporting), writing – original draft (supporting), writing – reviewing and editing (lead).

## Conflicts of Interest

The authors declare no conflicts of interest.

## Data Availability

Data sharing not applicable to this article as no datasets were generated or analysed during the current study.

## Appendix A

**TABLE A1:** Search terms used for academic literature.

Group 1: Fentanyl analogues	Group 2: Novel synthetic opioids
Fentanyl analog*	Benzenesulfonamide
Fentanyl analogue	U‐50488
Sufentanil	o amd
Alfentanil	O‐desmethyltramadol
Carfentanil	Etonitazene
Furanylfentanyl	U‐ 47700
Methylfentanyl	U‐47700
3‐Methylfentanyl	MT‐45
Acetylfentanyl	AH‐7921
Fluorofentanyl	4‐ANPP
Ocfentanil	Nortilidine
Acrylfentanyl	Isotonitazene
Cyclopropylfentanyl	Brorphine
Non‐pharmaceutical fentanyl	*Nitazene
Butyrfentanyl	Metonitazene
Butyrylfentanyl	U‐49900
α‐methylfentanyl	4‐anilino‐N‐phenethylpiperidine
Methoxyacetylfentanyl	*n*‐pyrrolidino‐etonitazene
Beta‐hydroxythiofentanyl	2‐methyl‐AP‐237
4‐fluorobutyrfentanyl	Clonitazene
Fluorobutyrfentanyl	Butonitazene
Despropionylfentanyl	AP‐238
Orthofluorofentanyl	Piperidylthiambutene
Methyl furanylfentanyl	Etazene (etodesnitazene)
Thiofentanyl	5‐amino‐ isotonitazene
Acryloylfentanyl	2F‐viminol
4‐Fluoroisobutyrfentanyl	Beta‐U10
4‐Fluorofentanyl	
2‐Fluorofentanyl	Group 3: Generic terms
Benzylfentanyl	Novel psychoactive substances
3‐Furanylfentanyl	NPS
Parafluorobutyrfentanyl	Novel synthetic opioids
Parafluorobutyrylfentanyl	NSO
Benzyl furanylfentanyl	
Benzoylbenzylfentanyl	
4‐Methoxyfentanyl	
Isopropyl furanylfentanyl	
